# Exploratory assessment of milk biomarkers during non‑antibiotic treatment in experimental *Staphylococcus warneri* goat mastitis

**DOI:** 10.1007/s11274-026-05052-3

**Published:** 2026-06-11

**Authors:** Ugonna Henry Uzoka, Richard Costa Polveiro, Jéssica Lobo Albuquerque Caldeira, Eugenie Youngo Tchokote, Nneka Victoria Ani, Emmanuel Ifeanyichukwu Ugwor, Abelardo Silva-Júnior, Maria Aparecida Scatamburlo Moreira

**Affiliations:** 1 Laboratory of Bacterial Diseases, Veterinary Department, Federal University of Viçosa, Viçosa, MG Brazil; 2 Department of Veterinary Medicine, College of Veterinary Medicine, Michael Okpara University of Agriculture Umudike, Umudike, Nigeria; 3 Faculty of Veterinary Medicine and Zootecnia, Federal University of Uberlandia, Uberlândia, Brazil; 4 Department of Theriogenology, Michael Okpara University of Agriculture, Umudike, Nigeria; 5 Department of Biochemistry, Federal University of Agriculture, Abeoku ta, Nigeria; 6 Institute of Biological and Health Sciences, Federal University of Alagoas, Maceió, Alagoas Brazil

**Keywords:** Dairy goats, N‑acetyl‑β‑D‑glucosaminidase, Milk mineral composition, Milk microbiota transplantation, Non‑antibiotic intramammary therapy

## Abstract

Conventional milk indicators such as somatic cell count (SCC) and pH have limited specificity for monitoring caprine mastitis, particularly under non-antibiotic treatment protocols. This exploratory study evaluated the temporal behavior of milk N-acetyl-β-D-glucosaminidase (NAGase), SCC, total bacterial count (TBC), pH, and selected electrolyte indicators (Na, K, and Na/K ratio) across sequential non-antibiotic phases, including milk microbiota transplantation (MMT), Resilience (washout), and 7-epiclusianone, in an experimental *Staphylococcus warneri* mastitis model in goats. Using a paired-udder longitudinal design in six goats, treated udders showed sustained sodium elevation, potassium depletion, and increased Na/K ratio, indicating persistent blood–milk barrier disturbance across phases, whereas pH and TBC showed more variable responses. NAGase activity correlated positively with SCC and sodium and negatively with potassium, consistent with its association with mammary epithelial injury and inflammation. These findings suggest that NAGase, together with Na, K, and the Na/K ratio, may help characterize biomarker dynamics during non-antibiotic treatment. Given the exploratory design and small sample size, these findings require validation in larger studies before routine application.

**Supplementary Information:**

The online version contains supplementary material available at 10.1007/s11274-026-05052-3.

## Introduction

Mastitis is one of the most important diseases limiting productivity and welfare in dairy goats, causing reduced milk yield, altered milk composition and increased culling risk (Akers and Nickerson 2011; Kamel and Bakry 2024; Novac and Andrei 2020). Subclinical forms are particularly problematic in caprine herds because they lack overt clinical signs, yet can persist for long periods and compromise both udder health and milk quality (Haggag et al. 2019). Coagulase‑negative staphylococci, including *Staphylococcus warneri*, are frequent etiological agents in goats and are increasingly recognized as true intramammary pathogens capable of establishing chronic infection and eliciting distinct neutrophil responses such as neutrophil extracellular trap (NET) formation (Caldeira et al. 2024).

Traditional mastitis diagnostics in ruminants rely on somatic cell count (SCC), the California Mastitis Test (CMT) and milk bacteriology, but these tools show important limitations when directly translated from cows to goats (Koop et al. 2011; Margatho et al. 2021). Goats are apocrine milk secretors, and normal caprine milk contains cytoplasmic particles and non‑infectious epithelial cells, resulting in higher baseline SCC and greater physiological variability influenced by breed, parity and stage of lactation (Smistad et al. 2025). Consequently, SCC thresholds validated in bovine milk have reduced specificity for caprine mastitis, and pH or electrical conductivity changes are often nonspecific and influenced by diet, management and sampling conditions(Nagahata et al. 2022; Tedde et al. 2019). In addition to SCC and CMT, total bacterial count (TBC) and milk pH are widely used indicators, but in goats they are often influenced by non‑infectious factors, further limiting their specificity for monitoring mastitis under field or experimental conditions (Abascal 2021). Beyond conventional indicators, mastitis in goats is associated with broad biochemical disturbances and altered enzymatic and antioxidant activity in milk, underscoring the need for additional biomarkers beyond SCC and pH (Novac and Andrei 2020). These constraints have motivated the search for more sensitive and goat‑adapted milk biomarkers capable of detecting subclinical intramammary inflammation and monitoring therapeutic responses.

N‑acetyl‑β‑D‑glucosaminidase (NAGase), a lysosomal enzyme released from damaged mammary epithelial cells and activated leukocytes, has emerged as a promising indicator of mammary tissue injury and intramammary inflammation (Miralles et al. 2024; Pyörälä 2003). Unlike SCC, which primarily reflects leukocyte influx, NAGase activity provides biochemical evidence of cellular damage and can rise early after blood–milk barrier disruption, sometimes preceding the major neutrophil influx in experimental models ( Leitner et al. 2004; Obara and Komatsu 1984). Studies in dairy goats and other ruminants have reported that NAGase activity in milk correlates with infection status, with infected udder halves showing significantly higher enzyme levels than non‑infected counterparts, and that NAGase can detect inflammation in cases where SCC is equivocal (Nagahata et al. 2022; Pratap et al. 2025). This positions NAGase as a candidate marker for both diagnosis and monitoring of mastitis, particularly in small ruminant systems where SCC interpretation is challenging (Maisi 1990; Stuhr and Aulrich 2010).

Beyond enzymatic indicators, mastitis is known to alter the ionic and mineral composition of milk through disruption of tight junctions and blood–milk barrier integrity (Li et al. 2021; Muhee et al. 2017). These changes typically include increased sodium and chloride and reduced potassium, with additional shifts in trace and minor elements such as copper, magnesium, zinc and molybdenum that may reflect both local epithelial damage and systemic acute‑phase or metabolic responses captured in milk (Singh et al. 2017).

At the same time, growing concern about antimicrobial resistance and drug residues in milk has stimulated interest in non‑antibiotic strategies for mastitis control, including microbiota‑based approaches and plant‑derived bioactives (Hoque et al. 2022; Maia et al. 2018). Milk microbiota transplantation aims to restore a beneficial mammary microbiome that competitively excludes pathogens, while phytochemicals such as 7‑epiclusianone, a natural compound with antimicrobial and antibiofilm properties, are being evaluated as intramammary treatments compatible with sustainable dairy production (de Souza et al. 2024; Flores‑Treviño et al. 2025; Niederwerder 2018; Peixoto et al. 2015). The successful implementation of such alternatives requires sensitive milk biomarkers capable of tracking both local mammary responses and systemic consequences over time. Taken together, these gaps highlight the need to describe how candidate milk biomarkers behave over time during non‑antibiotic treatment protocols for caprine mastitis. While individual biomarkers like NAGase and Na/K have been studied in bovine or static caprine mastitis, their combined temporal profiles under non-antibiotic therapies in goats represent a novel contribution, addressing gaps in sustainable mastitis management.

Therefore, this study aimed to explore the temporal behavior of milk NAGase, SCC, TBC, pH and mineral composition Na, K, Na/K ratio and trace elements across sequential non‑antibiotic treatment phases; milk microbiota transplantation (MMT), Resilience (washout) and 7‑epiclusianone in experimental *Staphylococcus warneri* goat mastitis.

We hypothesized that NAGase activity and Na/K ratio in milk would show stronger and more persistent associations with the non‑antibiotic treatment phases (MMT, Resilience and 7‑epiclusianone) than pH or TBC and, when combined with SCC and trace minerals, would better reflect blood–milk barrier integrity and mammary recovery than any single indicator alone.

## Materials and methods

### Experimental design and treatment protocol

A paired-udder design was employed in which the right udder half of each goat was experimentally infected with *S. warneri* and subjected to sequential non-antibiotic interventions, while the contralateral left udder served as an untreated control.

Experimental mastitis was induced using a *S. warneri* model as previously described in detail by Caldeira (2024), following the methodological framework of Peixoto et al. (2015). Briefly, the right udder of each goat was inoculated via intramammary infusion with 2 mL of a bacterial suspension (1.2 × 10⁸ CFU/mL). Establishment of infection was confirmed through bacteriological culture and routine mastitis indicators, including SCC, TBC, and CMT, in accordance with National Mastitis Council guidelines.

The infection model has been previously characterized with respect to microbiological dynamics and clinical evolution, and the present study focuses specifically on the temporal behavior of milk biomarkers within this established framework.

The experimental protocol spanned 20 days and comprised three sequential phases: MMT, a Resilience washout phase, and 7-epiclusianone treatment. During the MMT phase, the infected udder received daily intramammary infusion of 120 mL freshly collected donor goat milk. This was followed by a washout/Resilience phase during which no treatment was administered. Subsequently, 7-epiclusianone was applied once daily as an intramammary ointment (1.56 µg/mL in lanolin and petroleum jelly; 65 mL per dose, equivalent to 101.4 µg), with gentle massage to ensure distribution.

### Milk sampling

Milk samples were collected aseptically from both udder halves of six goats at predefined 2-day intervals throughout the experimental period, yielding 10 sampling time points on days 2, 4, 6, 8, 10, 12, 14, 16, 18 and 20. These sampling points spanned all phases of the protocol, with days 2–10 corresponding to the MMT phase, days 12–14 to the Resilience (washout) phase, and days 16–20 to the 7-epiclusianone phase.

Treatments were administered daily during the MMT and 7-epiclusianone phases, whereas no treatment was applied during the washout period. The apparent gaps between sampling days therefore reflect the predefined interval-based sampling schedule rather than interruptions in the experimental procedure.

At each time point, aliquots were collected for determination of N-acetyl-β-D-glucosaminidase (NAGase) activity, SCC, TBC, pH, and mineral composition. For the comparative analyses presented in this manuscript, however, SCC and TBC were included at a reduced analytical resolution (*n* = 14), whereas mineral-related variables were analyzed across the full repeated longitudinal dataset (*n* = 120). Samples were collected into sterile containers, transported on ice, aliquoted, and stored at − 80 °C until analysis.

### Enzymatic analysis

NAGase activity in milk samples was determined using a modified fluorometric plate assay (Hovinen et al. 2016). Briefly, 10 µL of each test sample was pipetted in duplicate into a black 96-well plate. Alongside the test samples, the following controls were included: Positive control: milk from a goat with clinical mastitis, Negative control: milk from a clinically healthy goat, treated sample: milk from the treated udder (right), Untreated control sample: milk from the contralateral udder (left), Blank: distilled water. Each well was incubated with 40 µL of the substrate solution, 4-methylumbelliferyl-N-acetyl-β-D-glucosaminide (4-MUAG; 2.25 mM, Sigma-Aldrich, M-2133). The enzymatic reaction was stopped by adding 150 µL of glycine buffer (0.2 M, pH 11).

The release of 4-methylumbelliferone (4-MU) was quantified in a fluorimeter at an excitation wavelength of 355 nm and an emission wavelength of 460 nm. The mean fluorescence values of duplicate wells were calculated and corrected by subtracting the mean fluorescence of the blank.

NAGase activity was calculated using the following equation:

$$\begin{array}{c}\mathrm{NAGase}\;\mathrm{activity}\;(\mathrm{sample})\;=\\\lbrack(\mathrm{Fluorescence_sample}-\mathrm{blank})\times\\\mathrm{NAGase}\;\mathrm{activity_Control}1\rbrack/\\(\mathrm{Fluorescence_Control}1-\mathrm{blank})\end{array}$$, where Control 1 was defined according to the standard calibration curve. Results were expressed as pmol of 4-MU released per minute per µL of milk at 20 °C.

Standard Curve - NAGase activity was quantified using a standard curve generated from the fluorescence of ten known concentrations of 4-methylumbelliferone (4-MU) ranging from 1 to 450 µM, prepared in a glycine buffer. The linear portion of this curve defined the lower and upper limits of quantification for the assay. To establish a reference value, the fluorescence of milk from a clinically mastitic goat (Control 1) was measured, corrected by subtracting the blank (distilled water), and divided by the slope of the standard curve, yielding the molarity of Control 1. This process was repeated in ten replicates at three independent time points to ensure accuracy. The resulting value was used as the calibration parameter for analyzing NAGase activity in all test samples.

###  Physicochemical analysis

SCC was determined by flow cytometry using an automated analyzer (NexGen Somacount FCM, Bentley Instruments, USA) at EMBRAPA Gado de Leite (Juiz de Fora, MG, Brazil). Samples were analysed in duplicate and results expressed as cells/mL.

TBC was determined with the Bactocount IBC system (Bentley Instruments, USA) and expressed as CFU/mL, using refrigerated samples preserved with Azidiol^®^ tablets.

Milk pH was measured using a calibrated benchtop pH meter equipped with automatic temperature compensation. The electrode was calibrated daily with pH 4.0, 7.0 and 10.0 standard buffers, and milk samples were equilibrated to 25 °C before analysis. Three replicate measurements were performed per sample and averaged.

### Elemental and mineral analysis

Milk mineral composition was evaluated by scanning electron microscopy coupled with energy-dispersive X-ray spectroscopy (SEM–EDS). Elements assessed included sodium (Na), potassium (K), calcium (Ca), chlorine (Cl), phosphorus (P), magnesium (Mg), iron (Fe), copper (Cu), zinc (Zn), molybdenum (Mo), and a composite carbon–nitrogen–oxygen (CNO) fraction representing the organic matrix. Given the exploratory nature of the study, SEM–EDS was used to obtain relative, comparative estimates of elemental composition across samples analyzed under standardized preparation and acquisition conditions, rather than absolute quantification equivalent to fully quantitative elemental methods.

Milk samples were prepared as thin films or dried droplets on appropriate substrates, dehydrated as needed, and examined under SEM with EDS detection operated under standardized and calibrated analytical conditions. For each sample, nine spectra were collected and averaged to improve measurement stability and generate semi-quantitative elemental estimates, reported in mg/L-equivalent values and interpreted comparatively across samples. The Na/K ratio was calculated as a derived variable to summarize electrolyte balance and blood–milk barrier perturbation.

### Data processing and statistical analysis

Raw data from NAGase, SCC, TBC, pH, and SEM–EDS were compiled and merged with the experimental metadata using the period identifiers, following the workflow established in our previous elemental biomarker study (Uzoka et al. 2025a). For the comparative analyses presented here, mineral-related variables were retained at the full repeated sample-level resolution, whereas SCC and TBC were included at a reduced analytical resolution, which accounts for the difference in n across variables.

All statistical analyses were performed using R version 4.3.0 (R Core Team 2023) with the following packages: tidyverse (v2.0.0) for data manipulation, ggplot2 (v3.4.2) for visualization, and rstatix (v0.7.2) for statistical tests. Because of the small sample size and non‑normal distribution of variables, non‑parametric methods were used and assessed by Shapiro-Wilk tests.

Between-phase comparisons were conducted using the Kruskal-Wallis rank sum test to evaluate differences across the three experimental phases. For variables showing significant omnibus effects (*p* < 0.05), post-hoc pairwise comparisons were performed using Dunn’s test with Benjamini-Hochberg false discovery rate (FDR) correction to account for multiple testing. Within-subject comparisons between control (left) and treated (right) udders were assessed using Wilcoxon signed-rank tests for paired samples. Effect sizes were calculated using the rank-biserial correlation coefficient (r).

Spearman rank correlation analysis was performed to explore associations among NAGase, SCC, TBC, pH and the 11 mineral variables (Na, K, Ca, Cl, P, Mg, Fe, Cu, Zn, Mo, CNO). For each pair of variables, Spearman’s ρ and corresponding p‑value were calculated. The Spearman method was chosen due to non-normal distribution and potential non-linear relationships. Correlations were considered strong when∣ρ∣>0.6 and statistically significant at *p* < 0.05, without further correction given the exploratory nature of this study; therefore, some associations, particularly those near the significance threshold, should be interpreted cautiously as they may represent false-positive findings. Results are presented as median and interquartile range (IQR) unless otherwise indicated. Median biomarker values in control and treated udder halves are summarized in Table 1, and phase‑dependent statistical comparisons are presented in Table 2.

**Table 1:** Median milk biomarker profiles in control (left) and treated (right) udder halves of goats with experimental *Staphylococcus warneri* mastitis across sequential non‑antibiotic treatment phases

Variable	n_Total	Median_Left	Median_Right	W_statistic	p_value	Effect_Size_r	Significance
Molybdenum	120	22.83	29.49833333	786.5	1.0553467033493242e-7	0.5630555555555555	***
Sodium	120	45.885000000000005	72.2225	840	4.751894793793368e-7	0.5333333333333333	***
Chlorine	120	174.76749999999998	223.265	879.5	1.3738856624954706e-6	0.5113888888888889	***
pH	118	6.8	7.1	1115	7.049849184029785e-4	0.3591954022988506	***
Na_K_Ratio	120	0.3015798499984591	0.5640709953917493	1207	0.001872023392922638	0.32944444444444443	**
NAGase	120	3112.42	4368.645	1402	0.036948385477633426	0.22111111111111115	*
Iron	120	1.7149999999999999	1.9125	1415	0.0435770303100084	0.2138888888888889	*
Copper	120	1.4725000000000001	1.92	1479	0.09252601918616739	0.17833333333333334	ns
Phosphorus	120	103.9275	111.29249999999999	1512	0.13130295902764008	0.16000000000000003	ns
Zinc	120	1.2925	1.33	1596	0.2854520512226182	0.11333333333333329	ns
TBC	14	90	237.5	6	0.3153024520817455	0.5	ns
Calcium	120	100.6725	102.325	1630	0.37365526339018595	0.09444444444444444	ns
Potassium	120	143.075	122.47	1941	0.46085783743921593	−0.078333333	ns
SCC	14	963.5	1967	8	0.5181824470434988	0.33333333333333337	ns
CNO	120	1493.4099999999999	1486.39	1877	0.6880366765715615	−0.042777778	ns
Magnesium	120	29.0525	28.7625	1833	0.8645531935134834	−0.018333333	ns

*SCC* somatic cell count, *TBC* total bacterial count, *NAGase* N‑acetyl‑β‑D‑glucosaminidase, *Na/K ratio* sodium‑to‑potassium ratio, *CNO* composite carbon–nitrogen–oxygen fraction. Phases: MMT (milk microbiota transplantation), Resilience (washout), 7‑Epi (7‑epiclusianone treatment). n represents the number of observations included in each analysis. Mineral variables were analyzed across the full repeated longitudinal dataset (*n* = 120), whereas SCC and TBC were analyzed within the same experimental framework but included here at a reduced analytical resolution (*n* = 14). Thus, differences in n reflect analytical structure rather than unexplained missing data

**Table 2:** Phase‑dependent differences in milk biomarkers during milk microbiota transplantation, Resilience and 7‑epiclusianone treatment in experimental caprine mastitis

Variable	*n*	H_statistic	df	p_value	Eta_squared	Significance
pH	106	18.65331404990388	2	8.901932963070432e-5	0.16168266067867845	***
Copper	108	10.611394431847389	2	0.0049632365742452115	0.08201328030330847	**
Na_K_Ratio	108	10.373853211009191	2	0.005589158132875832	0.07975098296199229	**
Sodium	108	7.824142031940198	2	0.01999903981221069	0.055468019351811414	*
Potassium	108	6.839509004417266	2	0.032720466755403826	0.04609056194683111	*
Magnesium	108	6.444330730915695	2	0.03986863456084583	0.042326959342054236	*
NAGase	108	5.562011552837248	2	0.0619761418777233	0.03392391955083093	ns
CNO	108	4.086544342507693	2	0.12960393002537743	0.01987185088102565	ns
Calcium	108	4.009068095065686	2	0.13472305561294695	0.019133981857768437	ns
SCC	14	2.207704654895664	1	0.13732285827442492	0.10064205457463866	ns
TBC	14	1.7999999999999972	1	0.1797124948790002	0.06666666666666643	ns
Phosphorus	108	2.18792048929663	2	0.3348876271918848	0.0017897189456821901	ns
Iron	108	1.6936289244361709	2	0.4287786492963608	−0.00291782	ns
Molybdenum	108	1.2046498260522784	2	0.5475371788475616	−0.007574764	ns
Zinc	108	0.5005637427708415	2	0.7785812923512437	−0.014280345	ns
Chlorine	108	0.1841560759861398	2	0.9120339734670064	−0.017293752	ns

*SCC* somatic cell count, *TBC* total bacterial count, * NAGase* N‑acetyl‑β‑D‑glucosaminidase, *Na/K ratio *sodium‑to‑potassium ratio, *CNO* composite carbon–nitrogen–oxygen fraction. Phases: MMT (milk microbiota transplantation), Resilience (washout), 7‑Epi (7‑epiclusianone treatment). Negative eta−squared values arise from bias−corrected estimators and should be interpreted as zero variance explained

Boxplots were generated to display the distribution of variables across experimental phases. Each boxplot shows the median (horizontal line within the box), interquartile range (IQR; box limits representing 25th and 75th percentiles), whiskers extending to 1.5 × IQR, and individual data points overlaid to illustrate data density and variability. Phase-specific color coding was applied: purple for MMT, teal for Resilience, and green for 7-Epiclusianone treatment. Statistical test results (Kruskal-Wallis p-values) are annotated on each plot. Full Dunn post‑hoc pairwise comparisons are provided in Supplementary Table S1, and strong significant Spearman correlations are presented in Supplementary Table S2.

Given the longitudinal sampling design, multiple observations were obtained from the same animals over time and from both udder halves. For the purposes of this exploratory analysis, observations were treated as independent to facilitate detection of temporal patterns and biomarker associations across treatment phases. However, this approach does not account for within-animal correlation and may lead to underestimation of variability and overestimation of statistical significance. Therefore, the reported p-values should be interpreted with caution.

The treatment phases were applied sequentially without randomization or parallel treatment groups; therefore, the design enables temporal assessment of biomarker dynamics but does not allow strict causal attribution to individual treatment phases.

## Results

### Overview of biomarker patterns across treatment phases

Across the sequential non‑antibiotic treatment phases, biomarker changes were generally more pronounced in the infected treated udders than in control halves, a pattern consistent with successful experimental mastitis induction and heterogeneous recovery dynamics. While some variables such as magnesium and pH appeared to normalize relatively quickly, others, notably sodium, potassium, Na/K ratio and copper, showed more prolonged alterations that extended beyond the 7‑epiclusianone treatment phase (Tables 1 and 2).

Differences in sample size between variables reflect differences in analytical resolution. Mineral parameters were analyzed across the full repeated longitudinal dataset, whereas SCC and TBC were included as a reduced set of harmonized observations within the same experimental framework. This distinction is consistent with the analytical structure previously reported for elemental biomarkers in the same goat mastitis model (Uzoka et al. 2025a).

### Copper and magnesium: systemic‑like versus transient responses

**Fig. 1:**
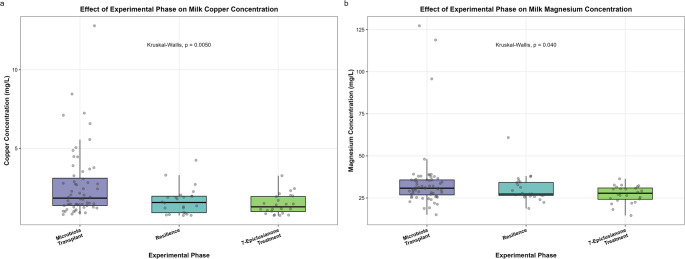
Phase-dependent changes in milk copper (**a**) and magnesium (**b**) concentrations during sequential non-antibiotic treatment

Milk Copper levels showed a gradual decline across the experimental timeline in both treated and control udders, with comparable trends observed between groups (Kruskal–Wallis χ² = 10.5, *p* = 0.005; Fig. 1a). Magnesium exhibited transient variation, with elevated values observed at specific intermediate time points, followed by a return toward baseline levels (Kruskal–Wallis χ² = 6.42, *p* = 0.04), with a marked bilateral peak at the end of the MMT phase followed by a rapid return to baseline in both udders (Fig. 1b). This transient spike may reflect a short‑lived systemic metabolic or stress response, distinct from the more persistent Na/K disturbance, since Mg normalized while Na, K and Na/K remained altered, particularly in treated udders.

### Sodium, potassium, Na/K ratio and pH: markers of barrier dysfunction

**Fig. 2:**
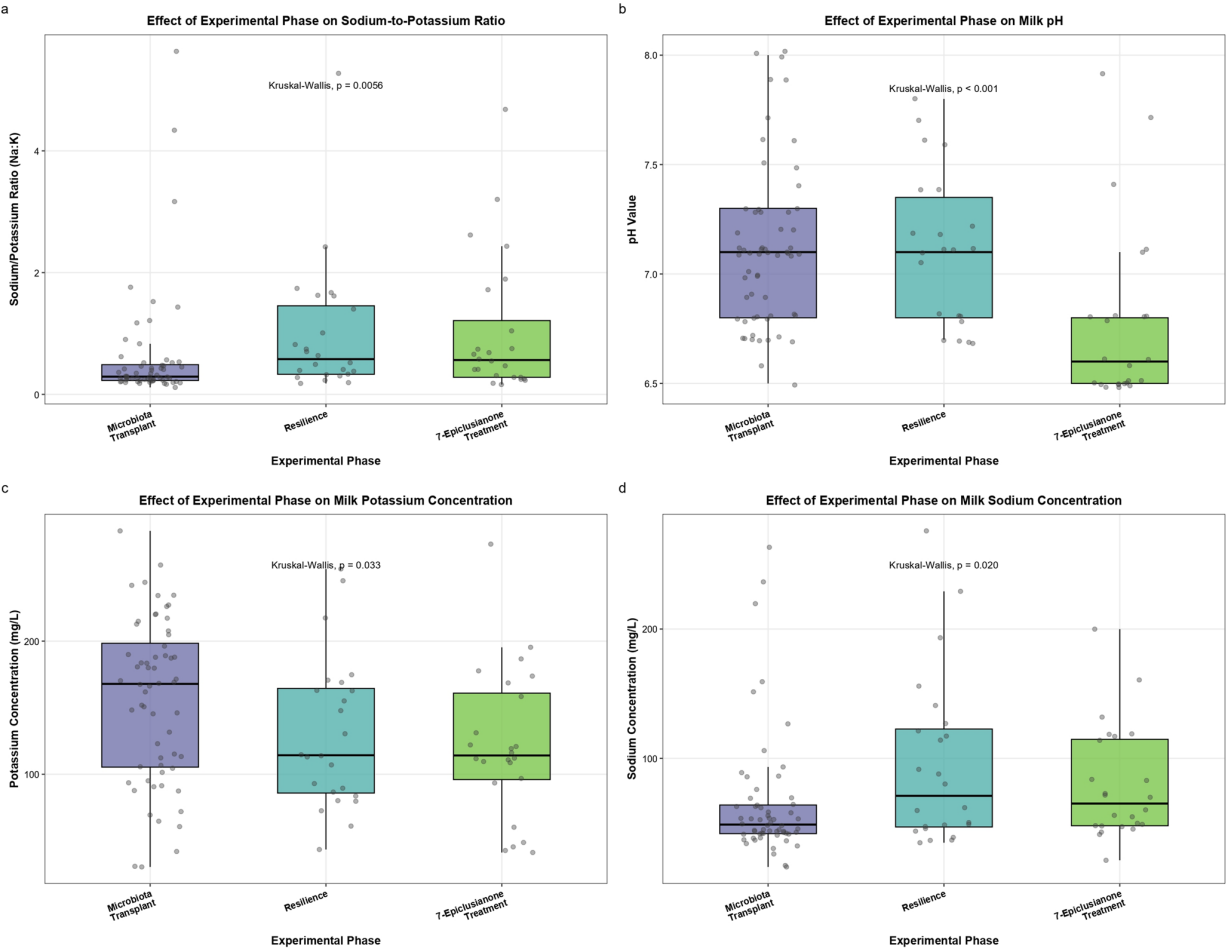
Phase-dependent changes in milk Na/K ratio (**a**), pH (**b**), potassium (**c**), and sodium (**d**) during sequential non-antibiotic treatment

The sodium‑to‑potassium (Na/K) ratio increased significantly from the MMT phase to the Resilience and 7‑epiclusianone phases in treated udders (Kruskal–Wallis χ² = 10.3, *p* = 0.0056), whereas control udders remained within a lower and narrower range (Fig. 2a). Elevated Na/K values in treated halves, often exceeding 1.0, suggest prolonged blood–milk barrier disturbance compatible with ongoing subclinical or clinical mastitis despite non‑antibiotic treatment.

Milk pH differed significantly between phases (Kruskal–Wallis χ² = 19.8, *p* = 8.9 × 10⁻⁵), with treated udders tending to show higher pH than controls during MMT and Resilience and both udders approaching more typical values during 7‑epiclusianone treatment (Fig. 2b). These patterns may reflect initial alkalinization associated with barrier opening and sodium–bicarbonate influx, followed by partial restoration of acid–base balance despite ongoing electrolyte imbalance.

Potassium, the predominant cation in healthy milk, showed higher median concentrations during the MMT phase compared with Resilience and 7‑epiclusianone, although substantial overlap in inter-quartile ranges indicated moderate variability (Kruskal–Wallis χ² = 6.82, *p* = 0.033; Fig. 2c). The decline from MMT to later phases appears consistent with progressive disruption of active ion transport and blood–milk barrier function, particularly in treated udders.

Sodium concentrations showed a progressive increase in the treated udder across the experimental timeline, with higher values observed during the MMT phase and persisting through the Resilience and 7-epiclusianone periods relative to the control (Kruskal–Wallis χ² = 7.85, *p* = 0.02), (Fig. 2d). In contrast, potassium levels displayed a decreasing trend in the treated udder over the same intervals. This sustained Na⁺ elevation in treated halves may indicate persistent blood–milk barrier dysfunction and inflammatory activity that was not fully resolved by the end of the non‑antibiotic treatment sequence. Pairwise phase comparisons for these biomarkers are detailed in Supplementary Table S1. In contrast, NAGase did not show statistically significant phase-dependent differences at the predefined threshold in this small dataset, despite trends towards higher values in treated udders.

Taken together, Figs. 1 and 2 suggest that while some parameters (e.g. magnesium, pH) tended to normalize relatively quickly, sodium, potassium, Na/K ratio and copper revealed more prolonged disruption of mammary homeostasis, especially in the treated udders, supporting their potential as exploratory milk‑based indicators of barrier integrity and systemic inflammatory adaptation.

Boxplots show concentrations (mg/L) in control (left) and treated (right) udder halves of goats with experimental *Staphylococcus warneri* mastitis across milk microbiota transplantation (MMT), Resilience (washout), and 7-epiclusianone treatment phases. Each box represents the median and interquartile range (IQR), whiskers extend to 1.5 × IQR, and points indicate individual observations. Kruskal–Wallis p-values are shown.

Boxplots represent measurements from control (left) and treated (right) udder halves of goats with experimental *Staphylococcus warneri* mastitis across milk microbiota transplantation, Resilience (washout), and 7-epiclusianone treatment phases. Each box shows the median and interquartile range (IQR), whiskers extend to 1.5 × IQR, and points represent individual observations. Kruskal–Wallis test p-values are indicated in each panel.

Heatmap of mean percent change in milk Na, K, Na/K ratio, Cu, Mg and pH relative to day 1 baseline across non-antibiotic treatment phases: milk microbiota transplantation (MMT), Resilience (washout) and 7-epiclusianone treatment for control (left) and treated (right) udder halves. Warmer colors indicate increases and cooler colors decrease relative to baseline.

### Temporal trajectories relative to baseline

**Fig. 3:**
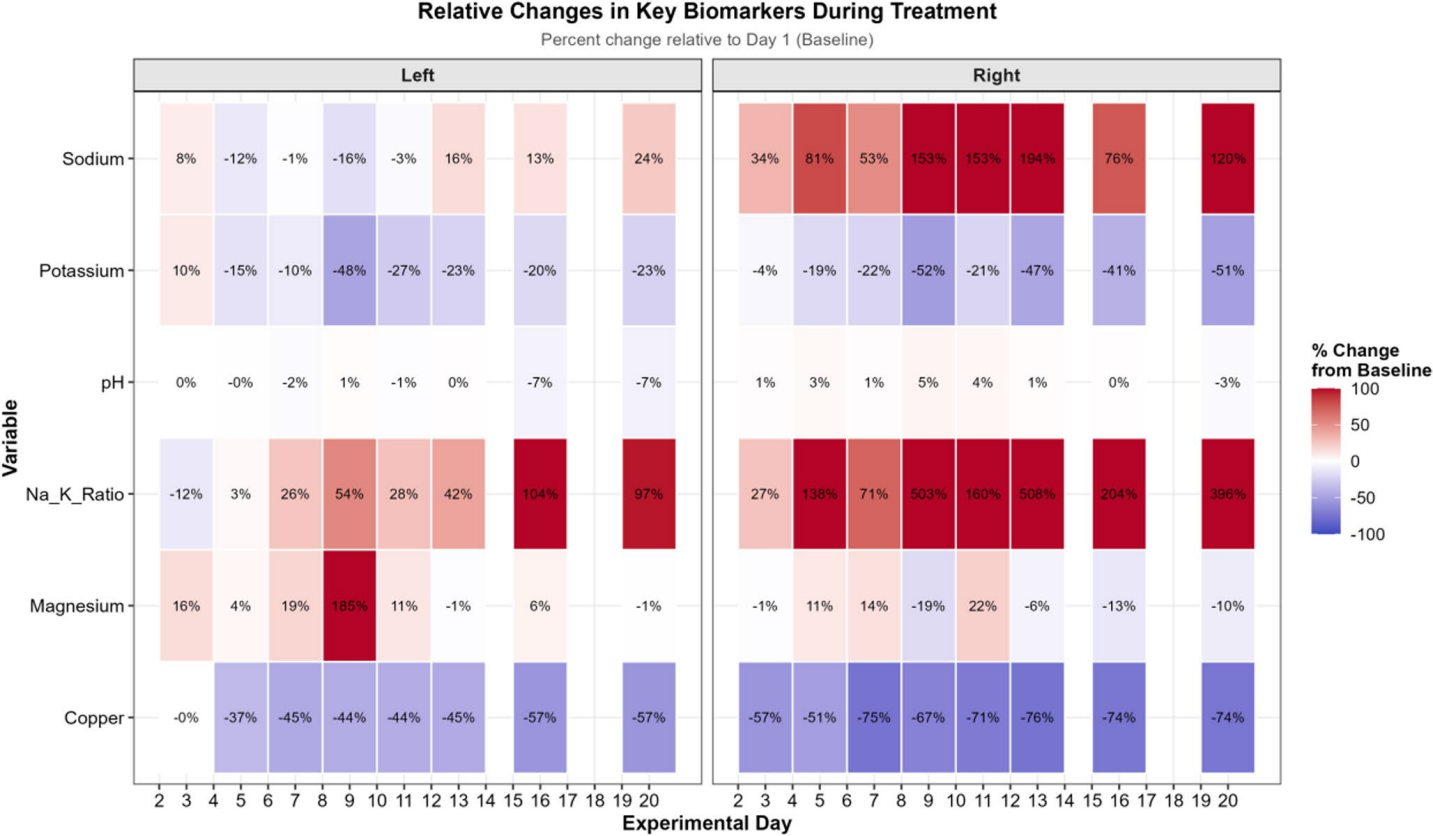
Percent change in selected milk biomarkers relative to baseline (day 1) across non-antibiotic treatment phases

Heatmap visualization of percent change relative to Day 1 baseline indicated that the treated udder experienced marked sodium elevations, reaching 81% above baseline by Day 5 and up to 194% during the late MMT phase, whereas the control udder varied only between − 16% and + 24% (Fig. 3). The Na/K ratio appeared to be the most sensitive integrated indicator, with the treated udder showing increases of 138–508% above baseline during Days 5–14, compared with more modest changes (3–104%) in the control (left half), while potassium declined in both udders but more markedly in the treated half. Copper concentrations decreased bilaterally by 37–76% across phases, which may reflect a sustained systemic acute‑phase response, whereas magnesium showed a transient bilateral elevation around the Resilience phase (Days 8–11), particularly in the control udder, followed by normalization. Milk pH changed by less than 7% in both udders despite pronounced electrolyte perturbations, and the continued elevation of sodium and Na/K ratio in the treated udder through the 7‑epiclusianone phase suggests incomplete restoration of blood–milk barrier integrity within the experimental timeframe.

### Correlation patterns among enzymatic, cellular and mineral biomarkers

**Fig. 4:**
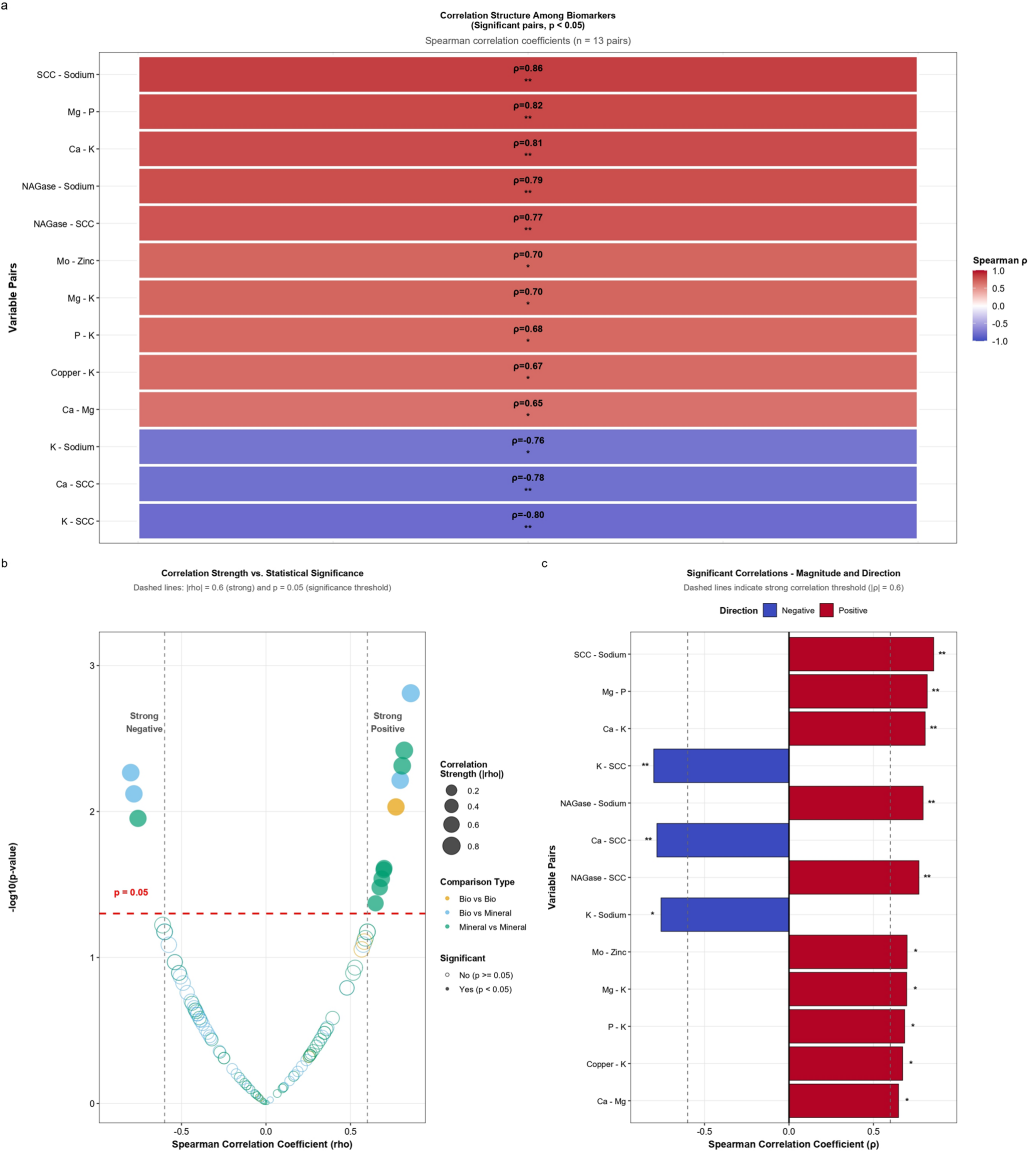
Correlation structure among milk biomarkers during non-antibiotic treatment. (**a**) Ranked heatmap of significant Spearman correlations (*p* < 0.05) among enzymatic, cellular, microbiological, and mineral variables. Color intensity represents the strength and direction of the correlation (red = positive, blue = negative). (**b**) Volcano plot showing the relationship between correlation strength (Spearman ρ) and statistical significance (–log₁₀ p-value). Dashed lines indicate thresholds for strong correlations (|ρ| ≥ 0.6) and statistical significance (*p* = 0.05). (**c**) Bar plot of significant correlations, ranked by magnitude, showing both direction (positive or negative) and strength. Asterisks indicate significance levels (*p < 0.05, **p < 0.01)

Correlation analysis revealed positive associations between NAGase, SCC, and sodium, and negative associations with potassium. The Na/K ratio showed strong alignment with sodium and inverse relationships with potassium, reflecting the underlying mathematical relationship between these variables (Fig. 4a, b,c). These associations were consistent across samples and suggest coordinated variation among enzymatic, cellular, and electrolyte indicators over time. The complete set of strong and statistically significant Spearman correlations is provided in Supplementary Table S2.Taken together, the strong positive correlations of SCC and NAGase with sodium, and their inverse relationships with potassium, support the interpretation that NAGase and Na/K ratio form part of an integrated biomarker set that describes blood–milk barrier disruption and mammary response under sequential non-antibiotic treatments. These correlations integrate data from both control and treated udders across all experimental phases, and thus reflect overall relationships among biomarkers rather than phase-specific effects.

### Phase-specific dynamics of key biomarkers

**Fig. 5:**
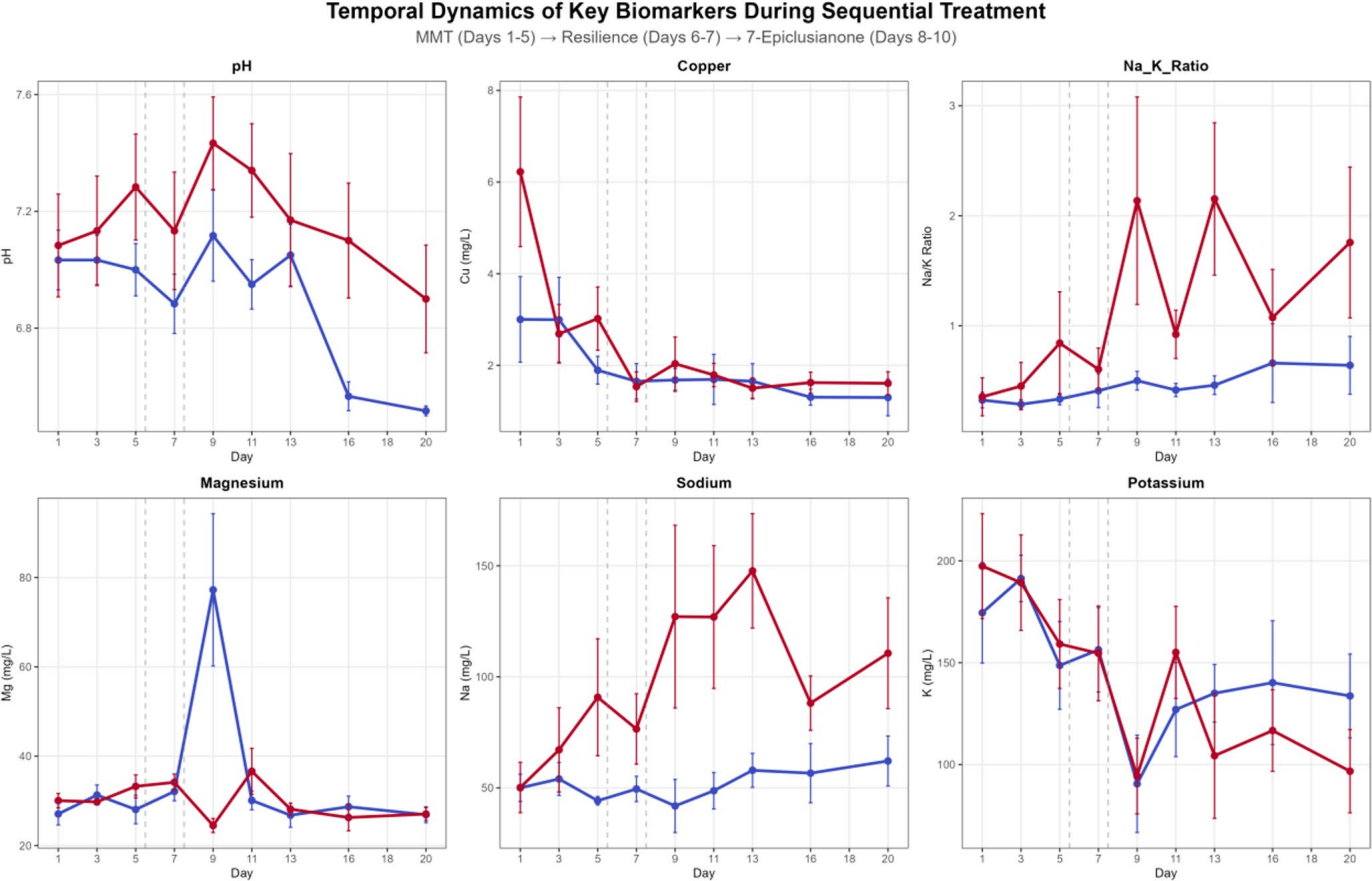
Temporal trajectories of key milk elemental biomarkers during non‑antibiotic treatment

Time‑series plots of the six key biomarkers (pH, Cu, Na, K, Mg and Na/K) suggested that treated udders generally showed larger deviations from baseline and from controls across the non‑antibiotic treatment phases (Fig. 5). Copper tended to decrease bilaterally and converge to similarly low levels by the end of the experiment (Supplementary Figure S1), while magnesium showed a short‑lived bilateral spike at the MMT–Resilience transition followed by apparent normalization (Supplementary Figure S2). Sodium and Na/K ratio in the treated udder rose sharply during MMT and often remained above control values through Resilience and 7‑epiclusianone, whereas potassium declined more in the treated than in the control half, with only partial recovery later in the trial (Supplementary Figure S3 and S4) and pH differences between udders were modest and gradually narrowed during 7‑epiclusianone (Supplementary Figure S5). Taken together, these trajectories suggest that Na, K and Na/K ratio may capture more persistent phase‑related disturbances in blood–milk barrier function, whereas Mg and pH appear to reflect more transient adjustments and Cu dynamics seem more compatible with a systemic acute‑phase–like response.

Time‑series plots of mean ± standard deviation for milk pH, Cu, Na, K, Mg and Na/K ratio in control (left) and treated (right) udders across 20‑day non-antibiotic phase treatment of *S.warneri* mastitis in goats. Vertical dashed lines indicate the boundaries of the milk microbiota transplantation (days 1–7), Resilience (days 8–13) and 7‑epiclusianone (days 16–20) phases (Kruskal-Wallis tests, *p* < 0.05). Each panel displays mean ± standard deviation (SD) for control (left udder, blue lines and points) and treated (right udder, red lines and points) measurements. Error bars show ± 1 SD.

## Discussion

This exploratory study investigated how a panel of candidate milk biomarkers behaves during sequential non‑antibiotic treatment phases in an experimental *S. warneri* mastitis model in goats, focusing on N‑acetyl‑β‑D‑glucosaminidase (NAGase), conventional indicators (SCC, TBC, pH) and a set of major and trace minerals. The overall patterns suggest that NAGase, together with Na, K and Na/K ratio, provides a more temporally resolved picture of mammary response and blood–milk barrier disturbance under milk microbiota transplantation (MMT), a Resilience (washout) period and 7‑epiclusianone treatment than any single conventional indicator alone.

### NAGase in relation to SCC and conventional indicators

NAGase activity in this model showed a positive association with SCC and sodium and an inverse relationship with potassium, a pattern consistent with its proposed role as an indicator of mammary epithelial damage and intramammary inflammation. Unlike SCC, which primarily reflects leukocyte influx, NAGase is considered to capture aspects of epithelial cell injury and enzymatic release into milk. Previous studies have reported that NAGase activity can increase following disruption of the blood–milk barrier and may rise before pronounced neutrophil infiltration in experimental mastitis models (Kumar et al. 2019; Leitner 2004; Nagahata et al. 2022; Rahularaj et al. 2020; Rani et al. 2020; Obara and Komatsu 1984; Yu et al. 2006). In addition, NAGase has been shown to correlate with infection status and may detect inflammatory changes in situations where SCC alone is difficult to interpret, particularly in small ruminants (Maisi 1990; Nagahata et al. 2022; Pratap et al. 2025; Stuhr and Aulrich 2010). These observations are consistent with broader evidence that mastitis in goats is associated with alterations in milk biochemical and enzymatic systems, including indicators such as NAGase (Novac and Andrei 2020). In the study, the alignment of NAGase with SCC and electrolyte changes suggests that it reflects overlapping but not identical aspects of the inflammatory response. Mechanistically, increased NAGase activity is likely associated with lysosomal enzyme release from damaged mammary epithelial cells and activated leukocytes, linking it to both tissue injury and cellular immune responses. This provides a biological basis for its association with SCC while supporting its interpretation as an indicator that extends beyond leukocyte enumeration.

Conventional indicators such as TBC and pH showed more variable and less consistent patterns across treatment phases. TBC exhibited substantial variability between animals and udder halves, which may reflect both biological heterogeneity and limitations of its use as a dynamic biomarker in small datasets. Milk pH showed relatively modest changes and tended to converge between treated and control udders over time, suggesting limited sensitivity to persistent alterations in mammary barrier function. These observations are consistent with previous reports indicating that pH and TBC can be influenced by non-infectious factors and may have limited specificity for intramammary infection when used alone (Kandeel et al. 2019; Abascal 2021; Koop et al. 2011; Nagahata et al. 2022; Tedde et al. 2019).

Taken together, these findings suggest that NAGase may provide complementary information to SCC, particularly when interpreted alongside electrolyte indicators such as Na, K and the Na/K ratio. However, given the small sample size and experimental design, these relationships should be interpreted as exploratory and require validation in larger studies.

### Electrolytes and Na/K ratio as indicators of barrier disturbance

From a regulatory perspective, the observed increase in sodium and decrease in potassium are consistent with disruption of tight junctions between mammary epithelial cells, leading to increased paracellular permeability and reduced selectivity of ion transport (Mabjeesh et al. 2007). Under normal conditions, active transport mechanisms maintain high potassium and low sodium concentrations in milk; however, during inflammation, breakdown of this barrier permits sodium influx and potassium efflux. The resulting elevation in Na/K ratio therefore represents an integrated functional indicator of blood–milk barrier dysfunction. This pattern is broadly consistent with reports of mastitis‑associated changes in milk composition and blood or milk leukocyte profiles in ruminants (Sarvesha et al. 2017; Vishnoi and Dang 2007).

Several studies in ruminants have linked increased milk Na and Na/K ratio and decreased K with mastitis or experimentally induced barrier opening, although the magnitude and consistency of changes in other ions such as Cl and electrical conductivity appear more context‑dependent (Fahmid et al. 2016; Guha et al. 2010). The present findings are broadly consistent with reports that combined Na increase and K decrease, and their ratio, are useful descriptors of udder infection and barrier dysfunction, particularly when interpreted together with SCC (El‑Zubeir et al. 2005; Haron et al. 2014; Nagahata et al. 2022). In this exploratory dataset, Na, K and Na/K ratio often discriminated treated from control udders more clearly than pH or TBC and tracked persistent disturbance into the 7‑epiclusianone phase, which may be relevant for monitoring incomplete recovery under non‑antibiotic protocols. Nevertheless, the limited number of animals and the experimental design caution against extrapolating absolute thresholds or diagnostic cut‑offs from these results.

### Trace minerals and possible systemic components

Trace and minor mineral dynamics appeared to capture additional aspects of the host response. This pattern is consistent with systemic regulation of copper during inflammatory responses, where redistribution toward hepatic synthesis of ceruloplasmin and other acute-phase proteins may reduce circulating and milk copper concentrations independently of local mammary processes as described in these studies Costa et al. 2010; Djoko et al. 2015; & Zheng et al. 2016). Such systemic control mechanisms may therefore contribute to the bilateral decline observed in this study. While altered copper concentrations have been reported during mastitis and systemic inflammation, the present data do not allow mechanistic conclusions about transport pathways; instead, they support the idea that milk Cu may reflect broader acute‑phase processes that overlap with local udder changes (Mungatana et al. 2011).

In this model, the synchronized and phase‑specific Mg increase, particularly in the control udder, may indicate a short-term metabolic or stress-related adjustment rather than sustained epithelial damage, potentially reflecting systemic regulation of magnesium in inflammatory or physiological stress conditions, including its role in cellular signaling and mitochondrial function. Transient shifts in Mg distribution have been described in acute stress or inflammatory states (Pilchova et al. 2017; Shahi et al. 2019). Other trace minerals, including Zn, Mo and Ca, showed more subtle or inconsistent changes, with some contributing to strong mineral–mineral correlations but without a clear, sustained divergence between treated and control udders. Together, these findings suggest that incorporating selected trace minerals may help distinguish between local barrier disruption and systemic components of the response, but further work is needed to identify robust patterns.

### Correlation structure and integrated biomarker panels

The correlation analysis, although exploratory and based on a small number of complete observations, provided additional insight into how different biomarker types may cluster. Strong positive correlations between SCC, NAGase and Na, and strong negative correlations involving K and Ca with SCC, are consistent with a coordinated inflammatory response in which leukocyte influx, epithelial damage and electrolyte shifts evolve together. At the same time, the predominance of strong mineral–mineral correlations (for example Mg–P and Ca–K) suggests that mineral homeostasis may be perturbed as part of broader systemic or metabolic adjustments, not only as a direct consequence of local inflammation.

NAGase is released from damaged epithelial cells and leukocytes due to lysosomal breakdown during inflammation (Nagahata et al. 2022), correlating with SCC (*r* = 0.77) and Na influx from barrier disruption. From a biomarker perspective, these correlation patterns support the notion that NAGase and Na/K ratio could be considered as components of an integrated multi‑marker panel, alongside SCC and selected minerals, for describing the dynamics of intramammary inflammation and blood–milk barrier function under non‑antibiotic treatment of mastitis. Importantly, the present data do not imply that NAGase or Na/K ratio should replace SCC or other established tests; instead, they point to potential complementarity and motivate further evaluation in larger, independent datasets and field conditions.

These mechanistic considerations suggest that the observed biomarker relationships are not merely statistical associations but reflect interconnected physiological processes involving epithelial integrity, inflammatory cell activity and systemic metabolic regulation. The convergence of NAGase, SCC and electrolyte shifts supports a coordinated response linking cellular damage, leukocyte infiltration and blood–milk barrier dysfunction, while trace mineral changes may reflect overlapping systemic adaptations.

### Methodological considerations and limitations

Several methodological features should be considered when interpreting these findings. The paired-udder, small-sample experimental design enabled detailed within-animal comparisons and high-resolution temporal assessment, but it also limits generalizability to larger populations and different pathogen contexts. In addition, repeated measurements within the same animals mean that observations are not fully independent; therefore, the present results should be interpreted as exploratory. In this respect, the current study extends our previous elemental biomarker analysis in the same goat mastitis model by integrating NAGase, conventional indicators, and electrolyte/mineral dynamics over time (Uzoka et al. 2025a).

The sequential non-antibiotic treatment protocol, informed by previous microbiota- and phytochemical-based studies (Uzoka et al. 2025a, b), provides a structured framework for evaluating biomarker dynamics, but it does not allow firm causal attribution to individual treatment phases. Accordingly, some observed patterns may reflect not only treatment-related influences, but also time-dependent processes, including the natural progression or resolution of infection.

Although the paired-udder design reduces inter-animal variability, the contralateral udder is an appropriate within-animal comparator, but not fully independent control, as systemic responses to infection or experimental handling may influence both udder halves. Furthermore, milk composition is subject to physiological variation due to factors such as stage of lactation, milk yield, milking interval, and diet; therefore, some changes observed over the 20-day experimental period may reflect these inherent influences rather than experimental effects alone.

Mineral analysis by SEM–EDS provides semi-quantitative, comparative estimates and is sensitive to sample preparation, acquisition conditions, and matrix-related effects; accordingly, these results are best interpreted as relative differences across samples and treatment phases rather than absolute mineral concentrations. In addition, the relatively large volume used for milk microbiota transplantation (120 mL per infusion) may represent a potential confounding factor, as it could influence milk composition through dilutional or mechanical effects independent of microbiological mechanisms. Likewise, the use of non-parametric statistical methods and an exploratory correlation approach was appropriate for the present dataset and study objectives, but the findings should still be regarded as hypothesis-generating rather than as establishing diagnostic thresholds or treatment efficacy.

Future studies incorporating larger sample sizes, randomized or parallel treatment designs, and fully quantitative analytical approaches, alongside integration of milk microbiome and clinical outcome data, are warranted to validate and extend these findings. Such work may help clarify the robustness of the observed NAGase-electrolyte relationships and the potential utility of Na/K ratio and selected trace minerals in mastitis monitoring.

## Conclusion

In this experimental *S. warneri* mastitis model in goats subjected to sequential non‑antibiotic treatments (MMT, Resilience and 7‑epiclusianone), the panel of milk biomarkers examined showed distinct temporal and phase‑related patterns. NAGase activity tended to align closely with SCC and with Na and K shifts, while pH and TBC showed more modest or variable changes. Sodium, potassium and the Na/K ratio, together with copper and magnesium, captured both persistent disturbances in blood–milk barrier function and transient systemic‑like responses that were not fully apparent from conventional indicators alone.

Overall, these exploratory findings suggest that NAGase, Na, K and Na/K ratio may offer a more nuanced description of mammary response and incomplete recovery under non‑antibiotic treatment protocols than single conventional markers when interpreted as part of a multi‑parameter panel. Given the small sample size and experimental context, these results should be considered hypothesis‑generating, and validation in larger field studies will be important before any routine diagnostic or monitoring application is proposed.

Supplementary Figures S1–S5 present time‑series plots (mean ± SD) for milk copper, magnesium, sodium, potassium and pH in control (left) and treated (right) udders across the milk microbiota transplantation (MMT), Resilience and 7‑epiclusianone phases, providing additional detail on biomarker trajectories during sequential non‑antibiotic treatment of experimental *S. warneri* caprine mastitis.

Full Dunn post‑hoc pairwise comparisons and significant Spearman correlations are provided in Supplementary Tables S1 and S2, respectively.

## Supplementary Information

Below is the link to the electronic supplementary material.

Supplementary Material 1 (DOCX 1.62 MB)

## Data Availability

All raw analytical data, processed datasets, metadata files, and R analysis scripts supporting this publication are available from the corresponding author upon reasonable request.
