# Supplementary material for: Exploratory assessment of milk biomarkers during non‑antibiotic treatment in experimental Staphylococcus warneri goat mastitis
Source: World J Microbiol Biotechnol. 2026 Jun 11;42(7):344. doi: 10.1007/s11274-026-05052-3 (PMC13253716; doi:10.1007/s11274-026-05052-3)
Supplement: Supplementary file 1 — Supplementary Material 1 (DOCX 1.62 MB) [file 11274_2026_5052_MOESM1_ESM.docx]

Supplementary files

Supplementary Table S1. Dunn post‑hoc pairwise comparisons for milk biomarkers across milk microbiota transplantation, Resilience and 7‑epiclusianone phases.

| Variable | Comparison | Z | P.unadj | P.adj |  |
| --- | --- | --- | --- | --- | --- |
| pH | 7-Epiclusianone Treatment - Microbiota Transplant | -4.116386185 | 3.848592431387089e-5 | 1.1545777294161267e-4 |  |
| pH | 7-Epiclusianone Treatment - Resilience | -3.466583884 | 5.271172303395071e-4 | 0.0015813516910185214 |  |
| pH | Microbiota Transplant - Resilience | -0.116375881 | 0.907354649543929 | 1 |  |
| Copper | 7-Epiclusianone Treatment - Microbiota Transplant | -2.944645965 | 0.0032332439626866693 | 0.009699731888060007 |  |
| Copper | 7-Epiclusianone Treatment - Resilience | -0.642876218 | 0.5203044219587016 | 1 |  |
| Copper | Microbiota Transplant - Resilience | 2.1762619172215807 | 0.029535681428490255 | 0.08860704428547077 |  |
| Na_K_Ratio | 7-Epiclusianone Treatment - Microbiota Transplant | 2.462086918958187 | 0.0138131187000802 | 0.0414393561002406 |  |
| Na_K_Ratio | 7-Epiclusianone Treatment - Resilience | -0.193550442 | 0.8465279056068408 | 1 |  |
| Na_K_Ratio | Microbiota Transplant - Resilience | -2.693423945 | 0.007072227871348238 | 0.021216683614044714 |  |
| Sodium | 7-Epiclusianone Treatment - Microbiota Transplant | 2.169060019431173 | 0.030078127513697286 | 0.09023438254109185 |  |
| Sodium | 7-Epiclusianone Treatment - Resilience | -0.11981694 | 0.904628162082499 | 1 |  |
| Sodium | Microbiota Transplant - Resilience | -2.312268655 | 0.020762882939903485 | 0.062288648819710454 |  |
| Potassium | 7-Epiclusianone Treatment - Microbiota Transplant | -2.238461127 | 0.025190999658287107 | 0.07557299897486132 |  |
| Potassium | 7-Epiclusianone Treatment - Resilience | -0.253458912 | 0.7999136078072895 | 1 |  |
| Potassium | Microbiota Transplant - Resilience | 1.9355197837179134 | 0.05292654333256868 | 0.15877962999770603 |  |
| Magnesium | 7-Epiclusianone Treatment - Microbiota Transplant | -2.499547438 | 0.012435204906297058 | 0.03730561471889118 |  |
| Magnesium | 7-Epiclusianone Treatment - Resilience | -1.138263645 | 0.2550104096251078 | 0.7650312288753234 |  |
| Magnesium | Microbiota Transplant - Resilience | 1.1390621644534926 | 0.25467722690704386 | 0.7640316807211316 |  |

^Na/K ratio, sodium‑to‑potassium ratio^

Supplementary Table S2. Strong and statistically significant Spearman correlations among milk biomarkers in goats during experimental *Staphylococcus warneri* mastitis non-antibiotic treatment phases

| Var1 | Var2 | rho | p_value | Significance |
| --- | --- | --- | --- | --- |
| SCC | Sodium | 0.8566683972885896 | 0.0015474724555037925 | ** |
| Magnesium | Phosphorus | 0.8181818181818181 | 0.003814920082550725 | ** |
| Calcium | Potassium | 0.806060606060606 | 0.0048620611019646025 | ** |
| Potassium | SCC | -0.800390911 | 0.005416207458694348 | ** |
| NAGase | Sodium | 0.7939393939393938 | 0.0060999233136969115 | ** |
| Calcium | SCC | -0.781631749 | 0.007570996323045855 | ** |
| NAGase | SCC | 0.7691256413612885 | 0.009306838448295092 | ** |
| Potassium | Sodium | -0.757575758 | 0.011143446799694262 | * |
| Molybdenum | Zinc | 0.6990913752320743 | 0.024470990757150354 | * |
| Magnesium | Potassium | 0.6969696969696969 | 0.02509667588225183 | * |
| Phosphorus | Potassium | 0.6848484848484847 | 0.028882797506732782 | * |
| Copper | Potassium | 0.6727272727272726 | 0.03304122254543776 | * |
| Calcium | Magnesium | 0.6484848484848484 | 0.042540127684489414 | * |
| Copper | Sodium | -0.612121212 | 0.05997214247725533 | ns |


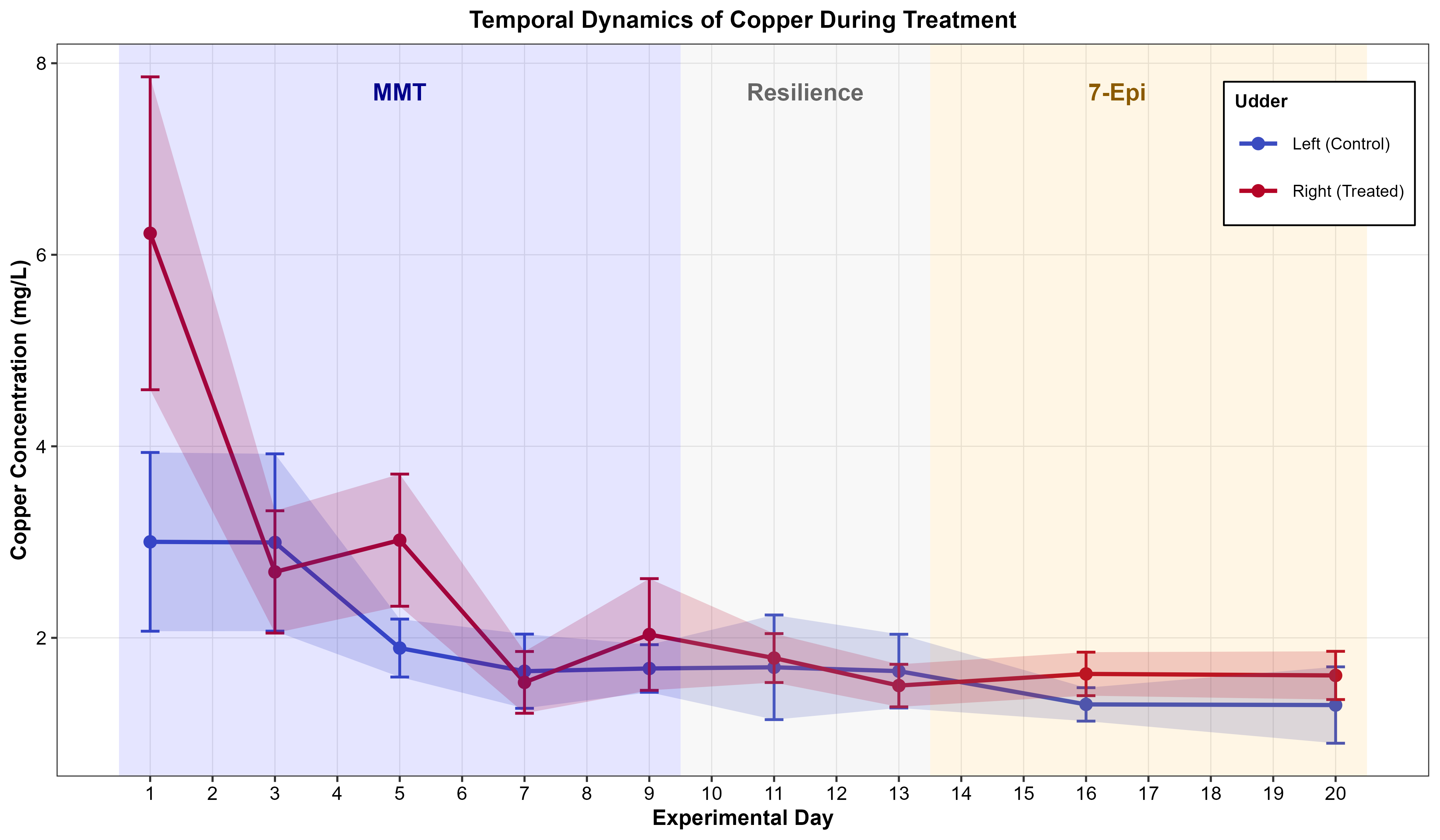


Supplementary Figure S1. Temporal dynamics of milk copper concentration during phases of non-antibiotic treatment of experimental *S. warneri* caprine mastitis


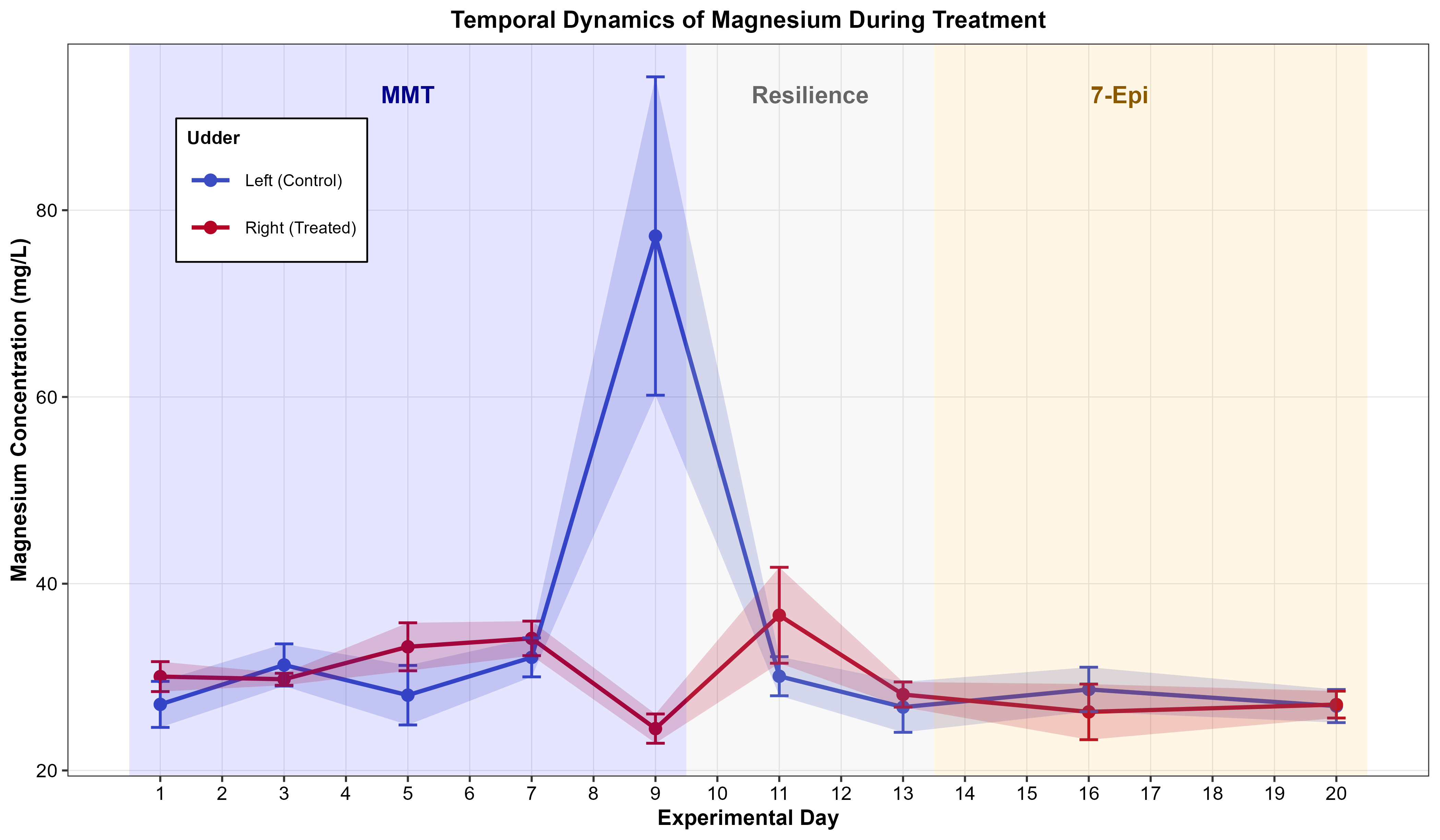


Supplementary Figure S2. Temporal dynamics of milk magnesium concentration during phases of non-antibiotic treatment of experimental *S. warneri* caprine mastitis


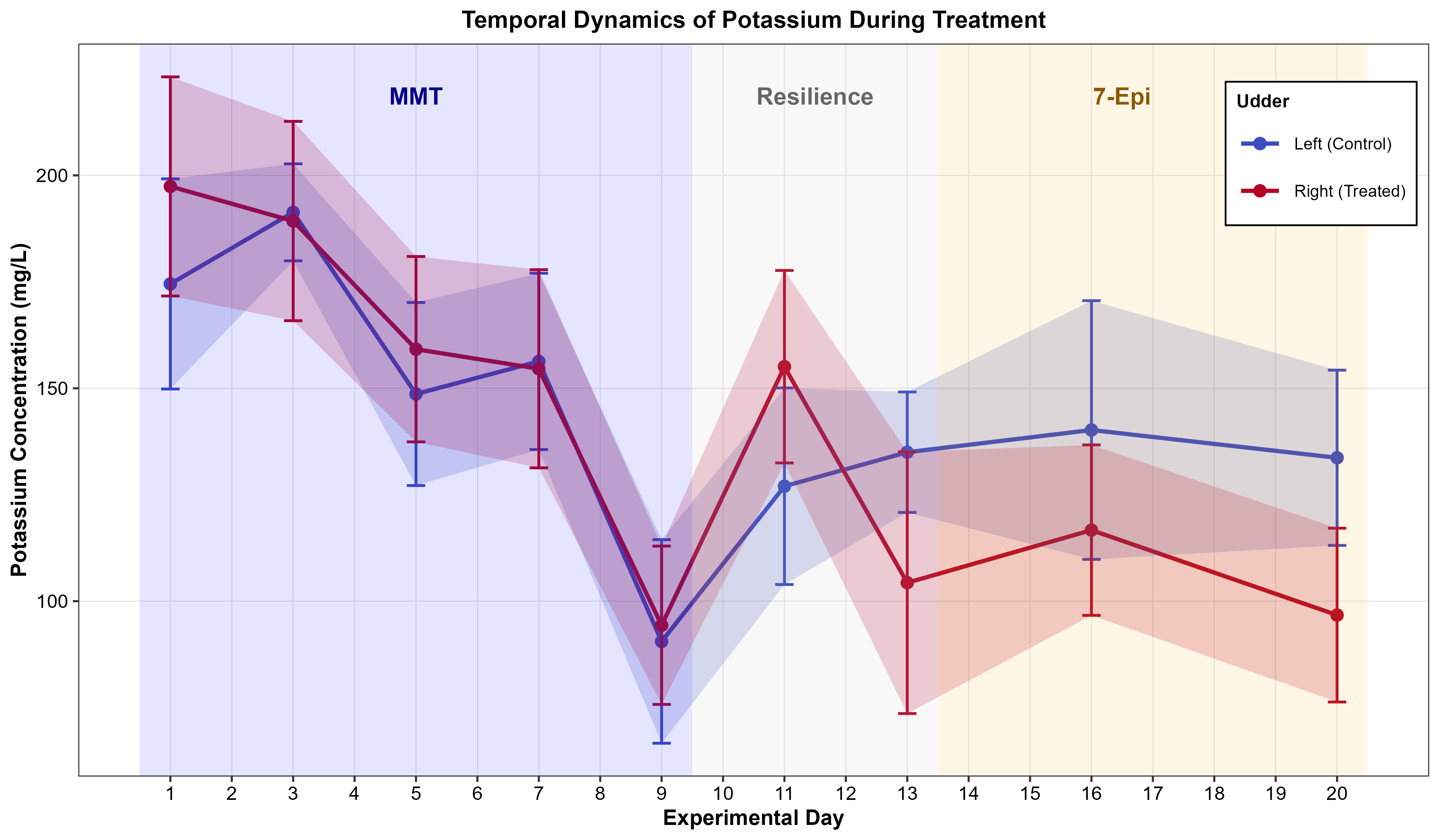


Supplementary Figure S3. Temporal dynamics of milk sodium concentration during phases of non-antibiotic treatment of experimental *S. warneri* caprine mastitis


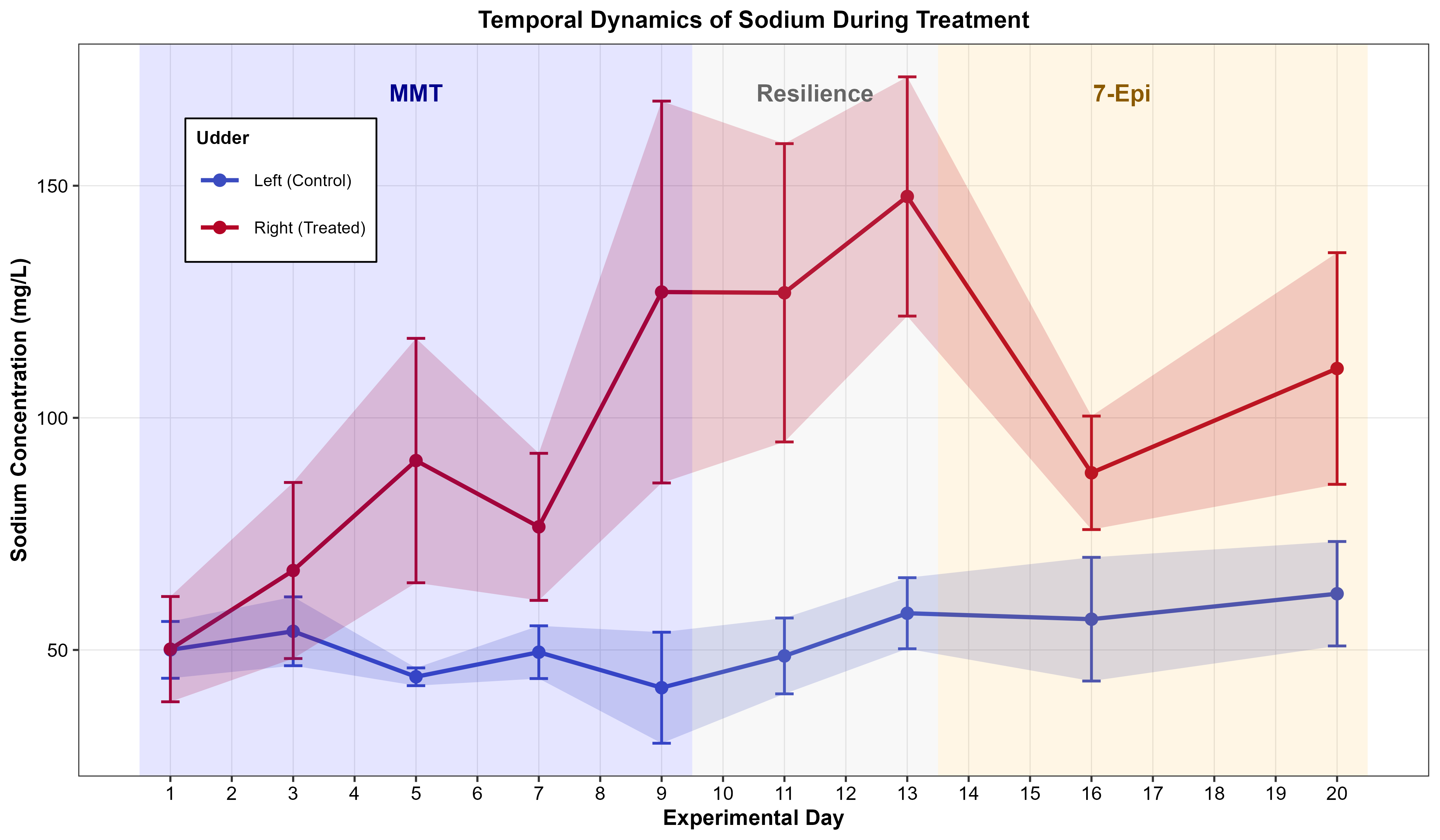


Supplementary Figure S4. Temporal dynamics of milk potassium concentration during phases of non-antibiotic treatment of experimental *S. warneri* caprine mastitis


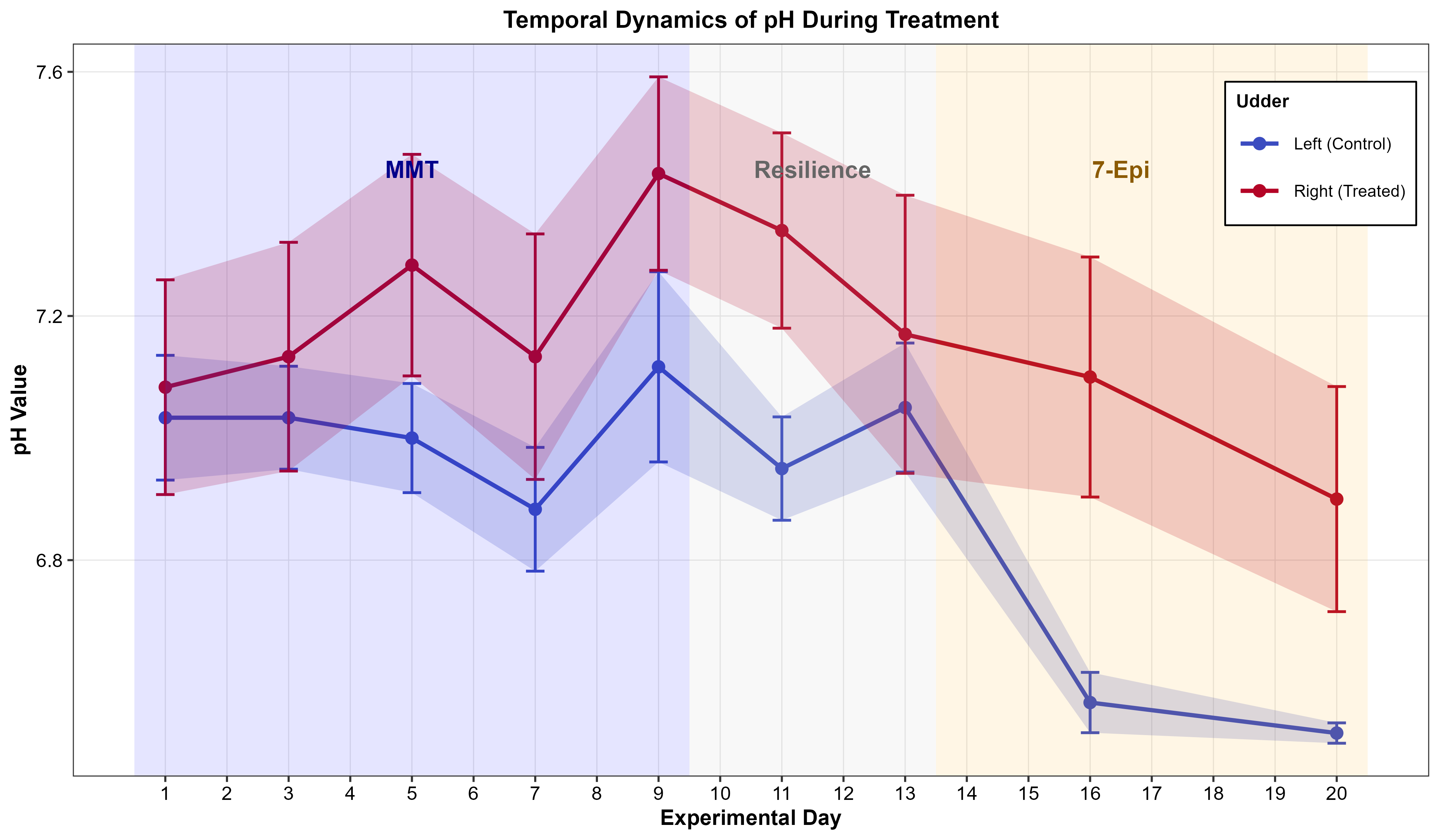


Supplementary Figure S5. Temporal dynamics of milk pH during phases of non-antibiotic treatment of experimental *S. warneri* caprine mastitis
